# Characteristics and outcomes of children 2–23 months of age with prolonged diarrhoea: A secondary analysis of data from the ‘Antibiotics for Children with Diarrhea’ trial

**DOI:** 10.7189/jogh.14.04196

**Published:** 2024-10-11

**Authors:** Irin Parvin, Abu Sadat Mohammad Sayeem Bin Shahid, Sharika Nuzhat, Mst Mahmuda Ackhter, Tahmina Alam, Md Farhad Kabir, Sharmin Khanam, Sunil Sazawal, Usha Dhingra, Judd L Walson, Benson O Singa, Karen L Kotloff, Samba O Sow, Naor Bar-Zeev, Queen Dube, Farah Naz Qamar, Mohammad Tahir Yousafzai, Karim Manji, Christopher P Duggan, Rajiv Bahl, Ayesha De Costa, Jonathon Simon, Per Ashorn, Tahmeed Ahmed, Mohammod Jobayer Chisti

**Affiliations:** 1 International Centre for Diarrhoeal Disease Research, Bangladesh (icddr,b), Dhaka, Bangladesh; 2 Centre for Public Health Kinetics, Delhi, India; 3 Department of International Health, Johns Hopkins Bloomberg School of Public Health, Baltimore, USA; 4 Department of Global Health, Epidemiology, Pediatrics and Medicine, University of Washington, Seattle, USA; 5 Kenya Medical Research Institute, Nairobi, Kenya; 6 Department of Pediatrics and Medicine, Center for Vaccine Development, University of Maryland School of Medicine, Baltimore, MD, USA; 7 Centre pour le Développement des Vaccins, Bamako, Mali; 8 International Vaccine Access Center, Johns Hopkins Bloomberg School of Public Health, Baltimore, Maryland; 9 Department of Maternal, Newborn, Child, and Adolescent Health and Aging, World Health Organization, Geneva, Switzerland; 10 Department of Pediatrics and Child Health, Aga Khan University, Karachi, Pakistan; 11 Department of Pediatrics and Child Health, Muhimbili University of Health and Allied Sciences, Dar es Salaam, Tanzania; 12 Division of Gastroenterology, Hepatology and Nutrition, Boston Children's Hospital, Boston, Massachusetts, USA; 13 Director General, Indian Council of Medical Research, Delhi, India; 14 Center for Child, Adolescent and Maternal Health Research, Faculty of Medicine and Health Technology, Tampere University, Tampere, Finland; 15 Department of Pediatrics, Tampere University Hospital, Tampere, Finland

## Abstract

**Background:**

Approximately 12% of all diarrhoeal episodes last for 7–13 days. As such, they are termed prolonged diarrhoea, and are associated with over two-thirds of all diarrhoeal deaths. Due to a lack of robust data, we aimed to evaluate a comparative background characteristics of young children with acute and prolonged diarrhoea, and their outcomes at day 90 follow-up.

**Methods:**

We performed a secondary analysis of data from the Antibiotics for Children with Diarrhea (ABCD) trial. Children aged 2–23 months were enrolled between July 2017 and July 2019 from seven Asian and sub-Saharan African countries. For this analysis, we divide diarrhoea into two categories: acute diarrhoea (duration <7 days) and prolonged diarrhoea (duration ≥7–13 days). We used logistic regression to observe baseline crude and adjusted associations and linear regression to compare post-discharge outcomes.

**Results:**

We analysed data on 8266 children, of whom 756 (9%) had prolonged diarrhoea and 7510 (91%) had acute diarrhoea. Pakistan had the highest proportion of children with prolonged diarrhoea (n/N = 178/1132, 16%), while Tanzania had the lowest (n/N = 12/1200, 1%). From an analysis that adjusted for sex, breastfeeding, nutritional status, clinical presentation, housing, water supply, sanitation, and country, we observed that presentation at a health facility with prolonged diarrhoea was associated with low age (2–12 months) (adjusted odds ratio (aOR) = 1.25; 95% confidence interval (CI) = 1.02, 1.53; *P* = 0.028), presence of three or more under-five children in the family (aOR = 1.54; 95% CI = 1.26, 1.87; *P* < 0.001), maternal illiteracy (aOR = 1.45; 95% CI = 1.21, 1.74, *P* < 0.001), moderate underweight (aOR = 1.25; 95% CI = 1.01, 1.55; *P* = 0.042) and pathogen (*Campylobacter*) (aOR = 1.27; 95% CI = 1.12, 1.44; *P* < 0.001). At day 90 follow-up, children with prolonged diarrhoea had significantly lower weight-for-age z-score compared to children with acute diarrhoea (−1.62, standard deviation (SD) = 1.11 vs −1.52, SD = 1.20; *P* = 0.032), as well as significantly higher frequency of hospital admission (6.1% vs 4.5%; *P* = 0.042).

**Conclusions:**

Prolonged diarrhoea was more common in children of younger age, those who were moderately underweight, those with *Campylobacter* in stool, those with three or more under-five children in a family, and those with illiterate mothers compared to those who had acute diarrhoea. Children with prolonged diarrhoea more often required hospitalisation during the three-month follow-up period compared to their counterparts.

Diarrhoea and its reciprocal relationship with malnutrition remain the leading causes of mortality and morbidity among young children globally, while their substantial burden in early childhood greatly impairs growth and development [1]. Estimates from 2019 place diarrhoea as the third leading cause of death among children younger than five years, with approximately half a million children (10% of 5.05 million under-five deaths) dying annually as a direct consequence of the condition [2].

Most diarrhoeal episodes are resolved within a week; however, a substantial proportion persists beyond this period and increases the risk of developing persistent diarrhoea. Such prolonged episodes of acute diarrhoea, defined as diarrhoea lasting 7–13 days, account for around 12% of all diarrheal episodes worldwide [3,4]. Importantly, prolonged diarrhoea in infancy increases the risk of developing persistent diarrhoea during the same hospitalisation. It is therefore worth noting that children undergoing severe and/or invasive diarrhoea are more likely to experience prolonged diarrhoea, compared to those experiencing mild to moderate diarrhoea [5,6]. In addition, if an acute episode of diarrhoea during hospitalisation progresses to prolonged diarrhoea, the risk of progression to persistent diarrhoea increases 6-fold [3]. The consequences of prolonged diarrhoea are associated with a number of morbidities in the form of increased rates of hospitalisation, nutritional derangements, and even deaths [7]. Over two-thirds of deaths associated with diarrhoea occur more than seven days after presentation, whereas about one-third of deaths occur after 21 days [8–10]. Yet while prolonged diarrhoea is common among children with severe acute malnutrition, its prevalence among children with moderate acute malnutrition remains unknown [11].

Although prolonged diarrhoea significantly affects children’s health, few studies have yet considered it as a distinct category of diarrhoea, which is why its epidemiology, aetiology, and nutritional impact are still poorly understood. Moore et al. [3] showed that maternal illiteracy (who did not complete primary education) and early weaning were risk factors for prolonged diarrhoea. Strand et al. [12] observed that being in a younger age group, not being breastfed, having a higher frequency of stool, and acquiring diarrhoea in the rainy season were risk factors for prolonged diarrhoea. Several previous studies identified age, breastfeeding status, nutritional status, stool frequency and diarrhoeal duration, month of enrolment/seasonality, and maternal illiteracy as risk factors for prolonged diarrhoea [3,12,13]. Here we compared the background characteristics of young children with acute and prolonged diarrhoea and their nutritional impact, as well as the related rates of hospitalisation during post-discharge follow-up at day 90.

## METHODS

### Study site

The seven Antibiotics for Children with Diarrhea (ABCD) field settings were established in diverse countries of South Asia (Bangladesh, India, and Pakistan) and sub-Saharan Africa (Kenya, Tanzania, Mali, and Malawi). Children aged 2–23 months with acute non-bloody diarrhoea were enrolled between July 2017 and July 2019 from 36 urban and rural outpatient settings.

### Study design and participants’ enrolment procedure

ABCD trial was a randomised, multi-country, multi-site, double-blind, placebo-controlled clinical trial which enrolled children aged 2–23 months presenting with acute non-bloody diarrhoea and meeting at least one of the following enrolment criteria: some or severe dehydration, and/or moderate wasting, and/or severe stunting (**Box 1**). The diarrhoeal classification was based on the self-reported duration of diarrhoea and was not investigated further. The detailed inclusion and exclusion criteria have been described in the published study protocol [19]. Anthropometric measurements were carried out after rehydration and/or stabilisation following the World Health Organization (WHO) guidelines. Primary caregivers or parents of the participants were interviewed, and relevant information such as socioeconomic and demographic characteristics, housing and environment situation, presenting features, health care seeking practices, vaccination status, and feeding practices of infant and young children were collected in a standard structured questionnaire after enrolment. The participants were followed up at day 90 since recruitment to determine their vital status, anthropometry, and hospitalisation status. All data were stored in a central database system. For this analysis, we retrieved secondary data of all enrolled children, irrespective of intervention status, from the ABCD trial database.

Box 1 **Operational definitions**

− Acute diarrhoea: passage of loose, watery stools, occurring three or more times in the last 24 hours of <7 days duration [14]. Prolonged diarrhoea: diarrhoea of a presumed infectious cause with acute onset lasting for 7–13 days [3].

− Severe diarrhoea: diarrhoea with moderate or severe dehydration [15]. Invasive diarrhoea: diarrhoea characterised by the presence of visible blood in the stools, usually associated with abdominal cramps and fever [16].

− Moderate stunting: length-for-age/height-for-age≤−2 SD and≥−3 SD of the median [17].

− Severe stunting: length-for-age/height-for-age<−3 SD of the median [17].

− Moderate underweight: weight-for-age<−2 SD and≥−3 SD of the median [17].

− Severe underweight: weight-for-age<−3 SD of the median [17].

− Moderate wasting: weight-for-length/weight-for-height≤−2 SD and≥−3 SD of the median [17]. Improved sources of drinking water: improved sources of drinking water include piped water, public taps, standpipes, tube wells, boreholes, protected dug wells and springs, rainwater, water delivered via a tanker truck or a cart with a small tank, and bottled water [18].

− Non-improved source of drinking water: unprotected well, unprotected spring and river/dam/lake/ponds/stream/canal/irrigation channel as non-improved source of drinking water of the household [18].

− Improved toilet facilities: include flush/pour flush toilets that flush water and waste to a piped sewer system, septic tank, pit latrine, or unknown destination; ventilated improved pit (VIP) latrines; pit latrines with slabs; or composting toilets [18].

− Non-improved toilet facility: flush to somewhere else or not known, pit latrine without slab/open pit, no facility/bush/field, hanging toilet were considered as non-improved toilet facility of household [18].

### Statistical analysis

We summarised the data using means/standard deviations (SDs) for continuous and frequencies/proportions for categorical variables, as well as visualised explanatory variables by countries using bar charts. χ^2^ test was used to determine the association between two categorical variables and *t-*test to assess the mean difference between the groups in cases where the distribution of data was symmetric. Determinants to be associated with prolonged diarrhoea were pre-selected based on biological plausibility and a literature review. They included sociodemographic variables (e.g. age, sex, maternal and household characteristics, site etc.) and clinical variables (e.g. nutritional status, clinical characteristics etc.).

We used logistic regression models to explore the association between baseline characteristics of prolonged and acute diarrhoea and adjusted the design effect of the clusters. We otherwise used linear regression models to compare the mean of anthropometric outcome and log-binomial regression to compare hospitalisation across the groups (prolonged diarrhoea vs acute diarrhoea) in surviving children, with the final model being adjusted for age, maternal education, number of under-five children in the family, baseline z-score, pathogen, and sites. We assessed the fitness of the logistic regression models using the Hosmer-Lemeshow test and the Pearson χ^2^ test. For the linear regression models, we evaluated their fitness per their R^2^ value and compared model fits using the Akaike information criterion and the Bayesian information criterion values. Multicollinearity was evaluated among the predictor variables using the variance inflation factor (VIF), with all VIF values being below the commonly accepted threshold of five (mean VIF = 1.45). To identify potential confounders, interaction terms were included in the regression models. We also checked and plotted residuals to identify any influential factors. We examined these residual plots to identify any outliers or influential observations (for the logistic regression models) or to ensure that the assumptions of linearity, homoscedasticity, and normality were met (for the linear regression models).

We conducted all analyses in Stata, version 14.0 (Stata Corp, College Station, TX, USA).  A *P*-value <0.05 indicated statistical significance.

## RESULTS

**Figure 1:**
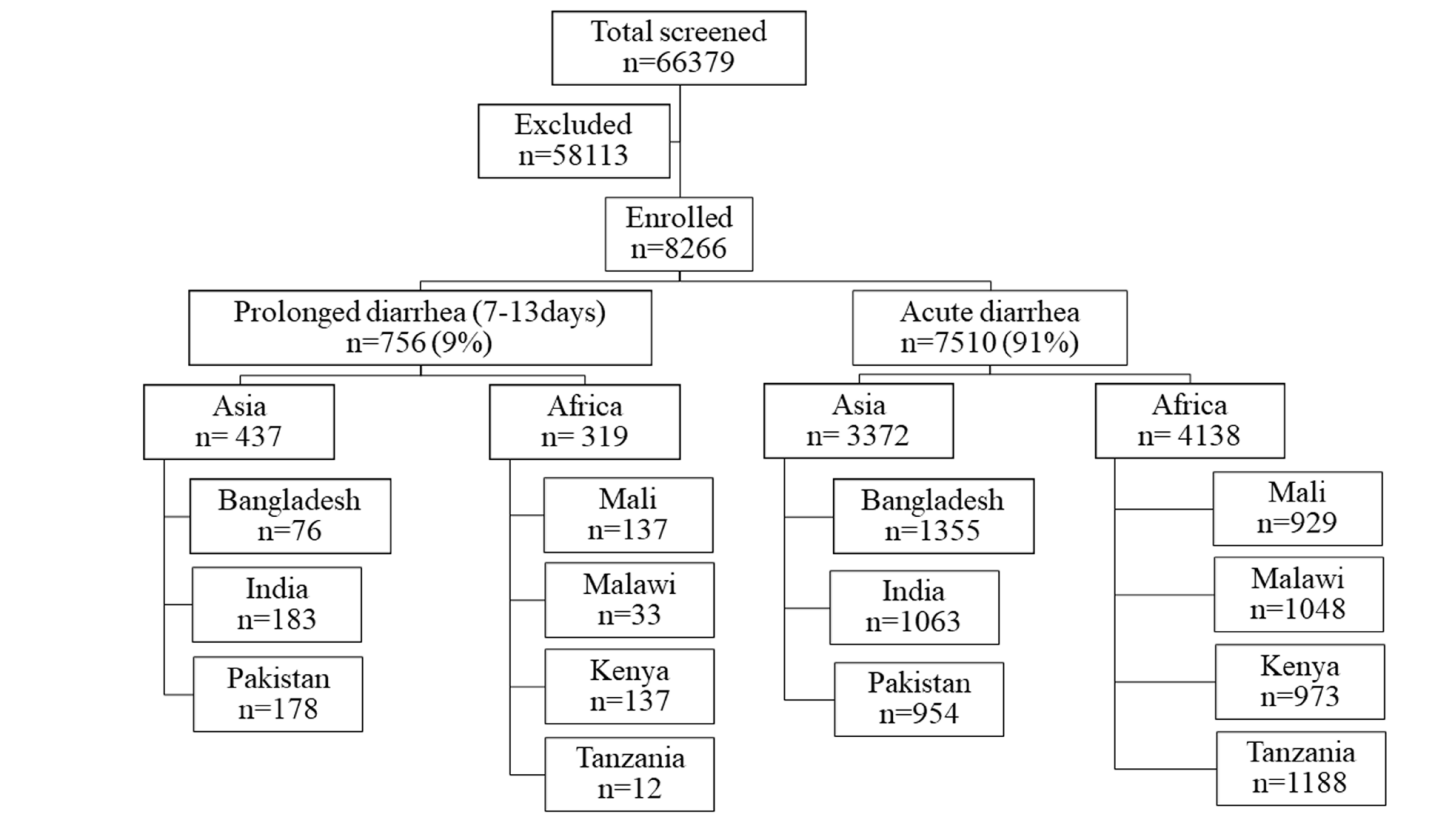
Study flowchart.

**Figure 2:**
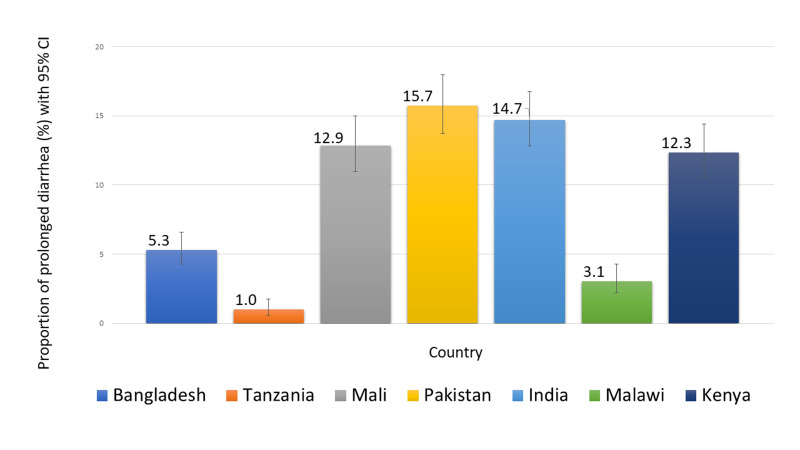
Site-specific proportion of prolonged diarrhoea.

A total of 66 379 children aged 2–23 months were screened for eligibility during the study period; 8266 fulfilled the enrolment criteria and were included in the analysis. Of these children, 756 (9%) had prolonged diarrhoea and 7510 (91%) had acute diarrhoea of <7 days duration. Among the seven study field sites, 3809 participants came from the Asian and 4457 from the African sites. In Asia, 12% study participants had prolonged diarrhoea, compared to 7% of the participants in Africa (**Figure 1**). Pakistan had the highest proportion of children (16%), while Tanzania had the lowest (1%) (**Figure 2**).

More than 55% (n/N = 452 out of 756 with prolonged diarrhoea and 4200 out of 7510 with acute diarrhoea) of the participants were 2–12 months old, and more than 50% were male (**Table 1**). Breastfeeding was found to be protective for prolonged diarrhoea, compared to acute watery diarrhoea (adjusted odds ratio (aOR) = 0.63; 95% confidence interval (CI) = 0.47, 0.83; *P* < 0.001) (**Table 3**). At baseline, children with prolonged diarrhoea more often had severe stunting, moderate wasting, and moderate or severe underweight than children with acute diarrhoea (**Table 1**). Compared to the children with acute diarrhoea, those with prolonged diarrhoea were more likely to have an illiterate mother, three or more under-five siblings in the family, or *Campylobacter* in stool, and were less likely to have dehydrating diarrhoea. We also found that prolonged diarrhoea was significantly associated with having unimproved toilet facilities, using non-piped water for drinking, and having a non-cemented floor in the house (**Table 1**, **Table 2****;** Table S1 and S2 in the **Online Supplementary Document**).

**Table 1:** Baseline characteristics of children aged 2–23 months with prolonged and acute diarrhoea

	Prolonged diarrhoea (n = 756), n (%)	Acute diarrhoea (n = 7510), n (%)	*P*-value
**Child characteristics**			
Sex			0.692
*Female*	353 (46.7)	3450 (45.9)	
*Male*	403 (53.3)	4060 (54.1)	
Age in months			0.041
*>12–23*	304 (40.2)	3310 (44.1)	
*2–12*	452 (59.8)	4200 (55.9)	
Breastfeeding status			<0.001
*Non-breastfed*	150 (19.8)	1000 (13.3)	
*Breastfed (exclusive and mixed)*	606 (80.2)	6507 (86.7)	
Nutritional status			
*Non-stunting*	475 (62.8)	4968 (66.2)	0.060
*Moderate stunting*	147 (19.4)	1418 (18.9)	0.718
*Severe stunting*	134 (17.7)	1115 (14.9)	0.036
*Non-wasting*	469 (62.1)	5059 (67.4)	0.003
*Moderate wasting*	286 (37.9)	2447 (32.6)	0.003
*No underweight*	356 (47.2)	4173 (55.6)	<0.001
*Moderate underweight*	284 (37.6)	2391 (31.9)	<0.001
*Severe underweight*	115 (15.2)	941 (12.5)	0.035
**Clinical presentation at enrolment**			
Frequency of loose stool in last 24 h			
*3–5 times*	273 (36.1)	2780 (37.0)	0.623
*6–10 times*	366 (48.4)	3635 (48.4)	0.996
*>10 times*	117 (15.5)	1095 (14.6)	0.507
Status of dehydration			<0.001
*No dehydration*	401 (53.0)	3355 (44.7)	
*Some/severe dehydration*	355 (47.0)	4155 (55.3)	
Pathogen			
*Campylobacter isolated in stool, n/N (%)*	348/636 (54.7)	2878/6054 (47.5)	<0.001

**Table 3:** Logistic regression model to see the independent association between prolonged and acute diarrhoea of children aged 2–23 months

Indicator	OR (95% CI)*	*P-*value	aOR (95% CI)†	*P*-value
Sex				
*Female*	ref		ref	
*Male*	0.97 (0.89, 1.06)	0.497	0.97 (0.86, 1.09)	0.592
Age category in months				
*>12–23*	ref		ref	
*2–12*	0.85 (0.74, 0.98)	0.029	1.25 (1.02, 1.53)	0.028
Breastfeeding status	re			
*Not breastfed*	ref		ref	
*Breastfed (exclusive and mixed)*	0.62 (0.43, 0.91)	0.014	0.63 (0.47, 0.83)	0.001
Stunting				
*Non-stunting*	ref		ref	
*Moderate stunting*	1.08 (0.77, 1.53)	0.648	1.01 (0.81, 1.27)	0.918
*Severe stunting*	1.26 (0.82, 1.92)	0.292	0.98 (0.79, 1.23)	0.892
Wasting				
*Non-wasting*	ref		ref	
*Moderate wasting*	1.45 (0.79, 2.65)	0.232	1.1 (0.79, 1.53)	0.591
Underweight				
*No underweight*	ref		ref	
*Moderate underweight*	1.39 (0.84, 2.32)	0.202	1.25 (1.01, 1.55)	0.042
*Severe underweight*	1.43 (0.83, 2.48)	0.198	1.2 (0.95, 1.53)	0.129
Dehydration status				
*None*	ref		ref	
*Some/severe*	0.71 (0.39, 1.3)	0.274	0.9 (0.71, 1.14)	0.381
Mother’s education				
*Primary and above (≥5)*	ref		ref	
*No education*	0.79 (0.48, 1.31)	0.359	1.45 (1.21, 1.74)	<0.001
*Below primary (<5)*	0.53 (0.32, 0.89)	0.016	1.53 (1.03, 2.25)	0.034
Number of under five children in household				
*One or two children*	ref		ref	
*Three or more children*	1.86 (1.25, 2.76)	0.002	1.54 (1.26, 1.87)	<0.001
Toilet				
*Not improved*	ref		ref	
*Improved*	0.62 (0.36, 1.04)	0.072	0.65 (0.43, 0.99)	0.045
Water supply				
*Not piped*	ref		ref	
*Piped*	0.63 (0.4, 0.98)	0.041	0.8 (0.58, 1.1)	0.174
Floor type				
*Not cemented*	ref		ref	
*Cemented*	0.64 (0.43, 0.96)	0.031	0.71 (0.56, 0.9)	0.005
Campylobacter				
*Not detected*	ref		ref	
*Detected*	1.33 (1.11, 1.61)	0.003	1.27 (1.12, 1.44)	<0.001

CI – confidence interval, aOR – adjusted odds ratio, OR – odds ratio, ref – reference

*Unadjusted ORs were calculated using simple logistic regression where outcome variable was diarrhoea type (0 = acute diarrhoea, 1 = prolonged diarrhoea).

†Adjusted ORs were calculated using multiple logistic regression where all the dependent variable given in the first column, were added in the model and also adjusted the potential design effect of the clusters (each country considered as a cluster).

**Table 2:** Baseline maternal and household characteristics of children aged 2–23 months with prolonged and acute diarrhoea

	Prolonged diarrhoea (n = 756), n (%))	Acute diarrhoea (n = 7510), n (%)	*P*-value
**Maternal characteristics**			
Maternal age in years			
*<20*	81 (10.7)	748 (10.0)	0.511
*20–29*	462 (61.1)	4702 (62.6)	0.417
*≥30*	213 (28.2)	2060 (27.4)	0.662
Maternal education			
*Primary and above (≥5)*	422 (56.0)	5199 (69.6)	<0.001
*No education (0)*	270 (35.8)	1761 (23.6)	<0.001
*Below primary (<5)*	62 (8.2)	512 (6.9)	0.159
Number of under 5 children in household			<0.001
*One or two children*	606 (80.2)	6626 (88.2)	
*Three and more children*	150 (19.8)	884 (11.8)	
**Household characteristics**			
Type of toilet facility			<0.001
*Not improved*	73 (9.7)	464 (6.2)	
*Improved*	683 (90.3)	7046 (93.8)	
Water supply			<0.001
*Not-piped*	317 (41.9)	2337 (31.1)	
*Piped*	439 (58.1)	5173 (68.9)	
Type of floor			<0.001
*Non-cemented*	173 (22.9)	1200 (16.0)	
*Cemented*	583 (77.1)	6308 (84.0)	

After adjusting for relevant covariates (i.e. those that were significant in the bivariate analysis, such as sex, nutritional status, breastfeeding, clinical presentation, housing, water supply, and sanitation) and the design effect of the cluster by the logistic regression model, we observed that children aged 2–12 months, those who were moderately underweight, those with *Campylobacter* in stool, those with three or more under-five children in the same family, and those with illiterate mothers had higher odds of developing prolonged diarrhoea. Conversely, being breastfed, having improved toilet facilities, and having cemented floors in the house were found to be protective factors for prolonged diarrhoea (**Table 3**).

**Figure 3:**
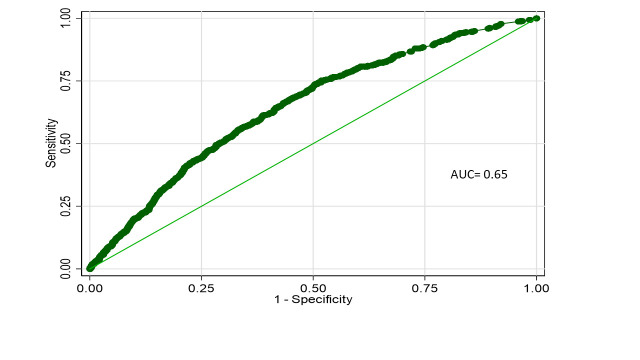
ROC curve for the final logistic regression model. AUC – area under the receiver operating characteristic curve, ROC – receiver operating characteristic curve.

When considered as a dichotomous variable, differences between the groups in anthropometric characteristics at baseline were comparable overall, except for the moderately underweight children (**Table 3**). Discriminatory capacity of the model to predict prolonged diarrhoea was moderate (0.65) (**Figure 3**). However, considering the anthropometric characteristics as a continuous variable, children with prolonged diarrhoea had significantly lower weight-for-age, weight-for-length, and length-for-age z-scores compared to their counterparts at baseline. However, at day 90 follow-up, children with prolonged diarrhoea had significantly lower weight-for-age z-scores compared to those who had acute diarrhoea (**Table 4**). The change in length-for-age z-score from day 1 to day 90 was 0.09 (SD = 0.61) in the prolonged diarrhoea group and −0.18 (SD = 0.60) in the acute diarrhoea group (risk difference = 0.04; 95% CI = −0.01, 0.08; *P* = 0.095) (**Table 5**). There was also a higher rate of hospitalisations (6.1%) by day 90 in the prolonged diarrhoea group compared to the acute diarrhoea group (4.5%) (relative risk = 1.30; 95% CI = 0.97, 1.76; *P* = 0.081) (**Table 5**). After age stratification, these effects were more pronounced among the younger age group, and there was no effect of intervention in the main trial (Table S3 in the **Online Supplementary Document**).

**Table 4:** Nutritional status of the study participants by duration of diarrhoea during the study period

	Prolonged diarrhoea, x̄ (95% CI)	Acute diarrhoea, x̄ (95% CI)	*P*-value
**At enrolment**			
Weight-for-age z-score	−1.91 (−2.00, −1.83)	−1.66 (−1.69, −1.63)	<0.001
Weight-for-length z-score	−1.29 (−1.38, −1.21)	−1.14 (−1.17, −1.12)	0.001
Length-for-age z-score	−1.66 (−1.75, −1.56)	−1.48 (−1.51, −1.45)	0.001
**At day 90**			
Weight-for-age z-score	−1.62 (−1.70, −1.53)	−1.52 (−1.54, −1.49)	0.032
Weight-for-length z-score	−0.94 (−1.02, −0.87)	−0.89 (−0.91, −0.86)	0.208
Length-for-age z-score	−1.74 (−1.83, −1.64)	−1.67 (−1.70, −1.64)	0.181

CI – confidence interval, x̄ – mean

**Table 5:** Anthropometric outcome and hospitalisation of the study participants enrolled at seven sites during the study period

	Prolonged diarrhoea (n = 756), x̄ (SD)	Acute diarrhoea (n = 7510), x̄ (SD)	*P*-value*	RD (95% CI)	RR (95% CI)	*P*-value
**Anthropometric outcome†**						
Change in 90-d Δ LAZ	−0.09 (0.61)	−0.18 (0.60)	<0.001	0.04 (−0.01, 0.08)		0.096
Change in 90-d Δ WAZ	0.29 (0.60)	0.17 (0.57)	<0.001	0.11 (0.07, 0.16)		<0.001
Change in 90-d Δ WHZ	0.35 (0.85)	0.29 (0.82)	0.045	0.12 (0.06, 0.17)		<0.001
**Hospitalisation‡**						
By day 90	46 (6.1)	335 (4.5)	0.042		1.30 (0.97, 1.76)	0.081

CI – confidence interval, LAZ – length-for-age z-score, RD – risk difference, RR – relative risk, SD – standard deviation, WAZ – weight-for-age z-score, WHZ – weight-for-length z-score, x̄ – mean, Δ – change

*t-test used for 90-d Δ LAZ, WAZ, and WHZ; χ^2^ used for hospitalisation.

**†**Linear regression adjusted for age, maternal education, number of under-five children in family, baseline z-score, and sites.

‡Log binomial regression adjusted for baseline z-score and site.

## DISCUSSION

We found that being aged 2–12 months, being moderately underweight, having *Campylobacter* in the stool, having more than three under-five children in the family, and having an illiterate mother were independent risk factors for prolonged diarrhoea. We also observed that children with prolonged diarrhoea were significantly more underweight at enrolment compared to those with acute diarrhoea; that they more frequently required hospitalisation during the follow-up period, and that they became less underweight at day 90 compared to enrolment, but the difference of weight change among children with acute diarrhoea compared to prolonged diarrhoea was actually less at day 90.

In resource-limited settings, prolonged episodes of acute diarrhoea can be crucial, not only because of the related morbidity, but also because of their associations with the overall burden of diarrhoea and malnutrition [20,21]. Recurrent diarrheal episodes are common, and the gut does not get sufficient time to recover between episodes [22]. Ultimately, the affected individuals become prone to develop other intercurrent illnesses such as respiratory infections, leading to recurrent hospitalisation. The prevalence of prolonged diarrhoea among the ABCD study patients was 9%, which is consistent with previous studies [3,7,11]. Although the proportion of prolonged diarrhoea compared to acute diarrhoea in this sample was lower, it contributed to significantly higher morbidity and hospitalisation, while also impacting the nutritional status among children under five years of age, which is consistent with a previous study [3]. It also contributed to the vicious cycle of diarrhoea and malnutrition [7,23].

Here we reported a significant protective association between breastfeeding and prolonged diarrhoea. The value and benefits of breastfeeding are well recognised, whereby the nutritional and bioactive component of breastmilk has optimal effects from the nutritional, physiological, and developmental viewpoints. The bioactive constituents of human milk include immunoglobulins, growth factors, microRNAs, and human milk oligosaccharides (HMOs). HMOs are non-digestible carbohydrates that have a valuable effect on shaping intestinal microbiota and modulating the immune response, thereby helping reduce infections by entero-pathogens. One of the most consistent findings of breastfeeding is that it protects children against diarrhoea [24], affecting not only its incidence, but also its severity [25]. These findings [24,25] are in line with the results of the current study.

Improved water, sanitation, and hygiene (WASH) practices (e.g. improved toileting with piped water under a cemented floor) are one of the most effective means of preventing diarrhoeal diseases in children which are known to contribute to reducing the global burden. They function by decreasing faecal-oral transmission of entero-pathogens, thus reducing the number of episodes and the duration of diarrhoea [26,27].

Regarding our other findings, having a higher number of under-five children in the family might lead to overcrowding, competition for the mother’s attention, and other factors that adversely affect general hygiene, consequently increasing the chance of contact with pathogens. Together with maternal illiteracy, these children were previously found to be more vulnerable to having diarrhoea [28].

The most prevalent form of childhood malnutrition is stunting or linear growth faltering. In low- and middle-income countries, diarrhoea and linear growth faltering continue to contribute to poor health. There is also a known bidirectional relationship between diarrhoea and malnutrition, whereby the risk of developing diarrhoea increases with poor nutritional status and *vice versa*, as assessed by anthropometric measurements. However, there are contrasting opinions among researchers regarding the effect of diarrhoea on childhood stunting [29]. For example, one study reported decreased linear growth following 1 or 2 months of an episode of diarrhoea [30], while another found this effect to be transient, as short intervals do not allow adequate time to achieve catch-up growth [31]. Catch-up growth may also be hampered if there is inadequate dietary intake, with further infection leading to decreased intake or another bout of diarrhoea [31]. Most studies related to diarrhoea and nutritional derangements did not address the potential role of prolonged diarrhoea in linear growth faltering, as compared to acute diarrhoea [29,30]. In this secondary analysis, we compared the effect of prolonged diarrhoea contrasting with acute diarrhoea. We did not find a gross growth faltering in relation to the clinical type of diarrhoea based on the duration used in our study. However, a population-based longitudinal study conducted at Matlab, Bangladesh, observed that the growth faltering of children is exacerbated by the presence of invasive diarrhoea [32].

A variety of interventions could be helpful in combatting diarrhoea; a prime example are exclusive breastfeeding and hygiene promotion, as they positively impact the nutritional status of the most susceptible children. Ideally, everyone should have access to adequate sanitation, safe drinking water, and hygiene promotion.

One of the limitations of our study is its inability to capture all cases of prolonged diarrhoea, as we were unable to follow up the cohort until the resolution of diarrhoea. This would have also allowed us to gain more insights into the enduring impact of prolonged diarrhoea on child health, especially on linear growth. We also lacked sufficient information on personal and microlevel hygiene or other cultural practices that could have impacted on the results.

## CONCLUSIONS

In our study, the children from Pakistan and Tanzania had the highest and lowest proportion of prolonged diarrhoea, respectively. Being older, being breastfed, following improved WASH practices, and having an educated mother served as protective factors for prolonged diarrhoea. Children with prolonged diarrhoea were also significantly associated with higher episodes of hospitalisation during subsequent months across all study sites, compared to those with acute diarrhoea. Our findings underscore the importance of early identification of children with prolonged diarrhoea for averting the long-term sequelae of hospitalisation, and they highlight a need for greater monitoring and clinical care in the management of prolonged diarrhoea during infancy to prevent morbidities in such children, particularly in resource-constrained settings.

## Additional material


Online Supplementary Document
