# Supplementary material for: Characteristics and outcomes of children 2–23 months of age with prolonged diarrhoea: A secondary analysis of data from the ‘Antibiotics for Children with Diarrhea’ trial
Source: J Glob Health. 2024 Oct 11;14:04196. doi: 10.7189/jogh.14.04196 (PMC11466500; doi:10.7189/jogh.14.04196)
Supplement: Online Supplementary Document [file jogh-14-04196-s001.pdf]

ONLINE SUPPLEMENTARY DOCUMENT

Characteristics and outcomes of children 2 to 23 months of age with prolonged diarrhoea: a secondary analysis from 'Antibiotics for Children with Diarrhea' trial

Irin Parvin[1] (https://orcid.org/0000-0002-6747-8223), Abu Sadat Mohammad Sayeem Bin Shahid[1] (https://orcid.org/0000-0002-7759-974X), Sharika Nuzhat[1], Mst. Mahmuda Ackhter[1], Tahmina Alam[1], Md. Farhad Kabir[1], Sharmin Khanam[1], Sunil Sazawal[2], Usha Dhingra[2], Judd L Walson[3,4], Benson O Singa[5], Karen L. Kotloff[6], Samba O Sow[7], Naor Bar-Zeev[8], Queen Dube[9], Farah Naz Qamar[10], Mohammad Tahir Yousafzai[10], Karim Manji[11], Christopher P. Duggan[12], Rajiv Bahl[13], Ayesha De Costa[9], Jonathon Simon[9], Per Ashorn[14,15], Tahmeed Ahmed[1], Mohammod Jobayer Chisti[1] (https://orcid.org/0000-0001-9958-3071)

Table S1: Age-stratified baseline characteristics of children aged 2-23 months with prolonged diarrhoea and acute diarrhoea

	Prolonged diarrhoea (%) (N= 756)			Acute diarrhoea (%) (N=7510)		
**Child characteristics**	**2-12m**	**>12-24m**	***P*-value**	**2-12m**	**>12-24m**	***P*-value**
**Sex**						
Female	208 (46)	145 (47.7)	0.650	1950 (46.4)	1500 (45.3)	0.337
Male	244 (54)	159 (52.3)	0.650	2250 (53.6)	1810 (54.7)	0.337
**Breast feeding status**						
Non-breastfed	393 (86.9)	213 (70.1)	<0.001	3880 (92.4)	2627 (79.4)	<0.001
Breastfeed (Exclusive and Mixed)	59 (13.1)	91 (29.9)	<0.001	318 (7.6)	682 (20.6)	<0.001
**Nutritional status**						
Non-stunting	315 (69.7)	160 (52.6)	<0.001	3055 (72.8)	1913 (57.9)	<0.001
Moderate stunting	68 (15)	79 (26)	<0.001	676 (16.1)	742 (22.5)	<0.001
Severe stunting	69 (15.3)	65 (21.4)	0.031	466 (11.1)	649 (19.6)	<0.001
Non-wasting	203 (44.9)	130 (42.8)	0.560	2288 (54.5)	1710 (51.7)	0.015
Moderate wasting	249 (55.1)	174 (57.2)	0.560	1912 (45.5)	1600 (48.3)	0.015
No underweight	230 (51)	126 (41.4)	0.010	2559 (61)	1614 (48.8)	<0.001
Moderate underweight	160 (35.5)	124 (40.8)	0.139	1191 (28.4)	1200 (36.3)	<0.001
Severe underweight	61 (13.5)	54 (17.8)	0.112	446 (10.6)	495 (15)	<0.001
**Clinical presentation at enrollment**						

Frequency of loose stool in last 24 hours						
3-5 times	163 (36.1)	110 (36.2)	0.973	1524 (36.3)	1256 (37.9)	0.139
6-10 times	208 (46)	158 (52)	0.100	2062 (49.1)	1573 (47.5)	0.176
>10 times	81 (17.9)	36 (11.8)	0.023	614 (14.6)	481 (14.5)	0.915
Status of dehydration						
No dehydration	232 (51.3)	169 (55.6)	0.249	1771 (42.2)	1584 (47.9)	<0.001
Some/Severe dehydration	220 (48.7)	135 (44.4)	0.249	2429 (57.8)	1726 (52.1)	<0.001
**Maternal characteristics**						
Maternal age in years						
<20 y	58 (12.8)	23 (7.6)	0.022	468 (11.1)	280 (8.5)	<0.001
20–29 y	263 (58.2)	199 (65.5)	0.044	2592 (61.7)	2110 (63.7)	0.071
≥30 y	131 (29)	82 (27)	0.547	1140 (27.1)	920 (27.8)	0.53
Maternal education						
No education (0)	162 (35.9)	108 (35.6)	0.938	932 (22.3)	829 (25.2)	0.004
Below primary (<5)	37 (8.2)	25 (8.3)	0.982	297 (7.1)	215 (6.5)	0.323
Primary and above (≥5)	252 (55.9)	170 (56.1)	0.950	2949 (70.6)	2250 (68.3)	0.034
No. of under 5 children in household						
One or Two children	359 (79.4)	247 (81.3)	0.537	3691 (87.9)	2935 (88.7)	0.292
Three and more children	93 (20.6)	57 (18.8)	0.537	509 (12.1)	375 (11.3)	0.292
**Household characteristics**						

Type of toilet facility						
Not improved	49 (10.8)	24 (7.9)	0.179	265 (6.3)	199 (6)	0.595
Improved	403 (89.2)	280 (92.1)	0.179	3935 (93.7)	3111 (94)	0.595
Water supply						
Not-piped	202 (44.7)	115 (37.8)	0.061	1299 (30.9)	1038 (31.4)	0.689
Piped	250 (55.3)	189 (62.2)		2901 (69.1)	2272 (68.6)	0.689
Type of floor						
Not-cemented	104 (23)	69 (22.7)	0.920	678 (16.1)	522 (15.8)	0.663
Cemented	348 (77)	235 (77.3)		3521 (83.9)	2787 (84.2)	0.663

Table S2: Baseline characteristics of children aged 2-23 months with prolonged diarrhoea and acute diarrhoea by country

	Bangladesh		India		Pakistan		Kenya		Mali		Malawi		Tanzania	
	Prolonged diarrhoea (%) (n=76)	Acute Diarrhoea (%) (n=1355)	Prolonged diarrhoea (%) (n=183)	Acute Diarrhoea (%) (n=1063)	Prolonged diarrhoea (%) (n=178)	Acute Diarrhoea (%) (n=954)	Prolonged diarrhoea (%) (n=137)	Acute Diarrhoea (%) (n=973)	Prolonged diarrhoea (%) (n=137)	Acute Diarrhoea (%) (n=929)	Prolonged diarrhoea (%) (n=33)	Acute Diarrhoea (%) (n=1048)	Prolonged diarrhoea (%) (n=12)	Acute Diarrhoea (%) (n=1188)
**Child characteristics**														
**Sex**														
Female	27(35.5)	582(43)	86(47)	496(46.7)	86(48.3)	469(49.2)	67(48.9)	464(47.7)	64(46.7)	433(46.6)	18(54.5)	448(42.7)	5(41.7)	558(47)
Male	49(64.5)	773(57)	97(53)	567(53.3)	92(51.7)	485(50.8)	70(51.1)	509(52.3)	73(53.3)	496(53.4)	15(45.5)	600(57.3)	7(58.3)	630(53)
Child Age														
2-12 months	53(69.7)	815(60.1)	101(55.2)	541(50.9)	97(54.5)	457(47.9)	85(62)	582(59.8)	89(65)	493(53.1)	20(60.6)	587(56)	7(58.3)	725(61)
12-24 months	23(30.3)	540(39.9)	82(44.8)	522(49.1)	81(45.5)	497(52.1)	52(38)	391(40.2)	48(35)	436(46.9)	13(39.4)	461(44)	5(41.7)	463(39)
**Breast feeding status**														
Non-breastfed	6(7.9)	79(5.8)	70(38.3)	398(37.4)	34(19.1)	165(17.3)	23(16.8)	134(13.8)	10(7.3)	89(9.6)	4(12.1)	47(4.5)	3(25)	88(7.4)
Breastfeed (Exclusive and Mixed)	70(92.1)	1276(94.2)	113(61.7)	665(62.6)	144(80.9)	789(82.7)	114(83.2)	839(86.2)	127(92.7)	838(90.4)	29(87.9)	1000(95.5)	9(75)	1100(92.6)
**Nutritional status**														
Non-stunting	44(57.9)	798(58.9)	88(48.1)	498(47)	86(48.3)	442(46.5)	118(86.1)	809(83.1)	103(75.2)	727(78.3)	25(75.8)	663(63.4)	11(91.7)	1031(86.8)
Moderate stunting	20(26.3)	314(23.2)	41(22.4)	276(26)	49(27.5)	244(25.7)	9(6.6)	107(11)	23(16.8)	141(15.2)	4(12.1)	208(19.9)	1(8.3)	128(10.8)
Severe stunting	12(15.8)	243(17.9)	54(29.5)	286(27)	43(24.2)	265(27.9)	10(7.3)	57(5.9)	11(8)	61(6.6)	4(12.1)	174(16.7)	0(0)	29(2.4)
Non-wasting	34(44.7)	662(48.9)	123(67.2)	676(62.8)	112(62.9)	615(64.5)	128(93.4)	889(91.4)	39(28.5)	288(31.0)	26(81.3)	887(85.0)	7(58.3)	1058(89.1)
Moderate wasting	42(55.3)	693(51.1)	60(32.8)	396(37.3)	66(37.1)	339(35.5)	9(6.6)	84(8.6)	98(71.5)	641(69.0)	6(18.8)	157(15.0)	5(41.7)	130(10.9)
No underweight	20(26.3)	451(33.3)	64(35)	412(38.8)	73(41)	347(36.4)	122(89.1)	849(87.3)	45(32.8)	374(40.3)	22(68.8)	722(69.2)	10(83.3)	1018(85.7)
Moderate underweight	47(61.8)	676(49.9)	79(43.2)	411(38.7)	65(36.5)	395(41.4)	10(7.3)	89(9.1)	73(53.3)	452(48.7)	8(25)	224(21.5)	2(16.7)	144(12.1)
Severe underweight	9(11.8)	228(16.8)	40(21.9)	240(22.6)	40(22.5)	211(22.1)	5(3.6)	35(3.6)	19(13.9)	103(11.1)	2(6.3)	98(9.4)	0(0)	26(2.2)
**Clinical presentation at enrollment**														

	Bangladesh		India		Pakistan		Kenya		Mali		Malawi		Tanzania	
	Prolonged diarrhoea (%) (n=76)	Acute Diarrhoea (%) (n=1355)	Prolonged diarrhoea (%) (n=183)	Acute Diarrhoea (%) (n=1063)	Prolonged diarrhoea (%) (n=178)	Acute Diarrhoea (%) (n=954)	Prolonged diarrhoea (%) (n=137)	Acute Diarrhoea (%) (n=973)	Prolonged diarrhoea (%) (n=137)	Acute Diarrhoea (%) (n=929)	Prolonged diarrhoea (%) (n=33)	Acute Diarrhoea (%) (n=1048)	Prolonged diarrhoea (%) (n=12)	Acute Diarrhoea (%) (n=1188)
Frequency of loose stool in last 24 hours														
3-5 times	3(3.9)	121(8.9)	33(18)	201(18.9)	56(31.5)	361(37.8)	79(57.7)	591(60.7)	84(61.3)	507(54.6)	13(39.4)	564(53.8)	5(41.7)	435(36.6)
6-10 times	33(43.4)	648(47.8)	99(54.1)	569(53.5)	99(55.6)	479(50.2)	56(40.9)	371(38.1)	53(38.7)	417(44.9)	19(57.6)	456(43.5)	7(58.3)	695(58.5)
>10 times	40(52.6)	586(43.2)	51(27.9)	293(27.6)	23(12.9)	114(11.9)	2(1.5)	11(1.1)	0(0)	5(0.5)	1(3)	28(2.7)	0(0)	58(4.9)
Status of dehydration														
No dehydration	60(78.9)	1003(74)	86(47)	533(50.1)	123(69.1)	688(72.1)	16(11.7)	71(7.3)	109(79.6)	720(77.5)	5(15.2)	251(24)	2(16.7)	89(7.5)
Some/Severe dehydration	16(21.1)	352(26)	97(53)	530(49.9)	55(30.9)	266(27.9)	121(88.3)	902(92.7)	28(20.4)	209(22.5)	28(84.8)	797(76)	10(83.3)	1099(92.5)
**Maternal characteristics**														
Maternal age in years														
<20 y	18(23.7)	199(14.7)	6(3.3)	28(2.6)	14(7.9)	59(6.2)	16(11.7)	91(9.4)	20(14.6)	129(13.9)	7(21.2)	173(16.5)	0(0)	69(5.8)
20–29 y	43(56.6)	865(63.8)	134(73.2)	785(73.8)	99(55.6)	552(57.9)	86(62.8)	613(63)	73(53.3)	498(53.6)	17(51.5)	642(61.3)	10(83.3)	747(62.9)
≥30 y	15(19.7)	291(21.5)	43(23.5)	250(23.5)	65(36.5)	343(36)	35(25.5)	269(27.6)	44(32.1)	302(32.5)	9(27.3)	233(22.2)	2(16.7)	372(31.3)
Maternal education														
No education (0)	15(19.7)	255(18.9)	72(39.6)	404(38)	106(59.6)	561(59)	2(1.5)	7(0.7)	74(54)	469(50.9)	0(0)	11(1.1)	1(8.3)	54(4.6)
Below primary (<5)	9(11.8)	185(13.7)	17(9.3)	78(7.3)	16(9)	61(6.4)	8(5.8)	44(4.6)	10(7.3)	34(3.7)	2(6.3)	92(8.8)	0(0)	18(1.5)
Primary and above (≥5)	52(68.4)	909(67.4)	93(51.1)	580(54.6)	56(31.5)	329(34.6)	127(92.7)	913(94.7)	53(38.7)	418(45.4)	30(93.8)	938(90.1)	11(91.7)	1112(93.9)
No. of under 5 children in household														
One or Two children	76(100)	1340(98.9)	145(79.2)	889(83.6)	135(75.8)	744(78)	114(83.2)	838(86.1)	93(67.9)	660(71)	31(93.9)	999(95.3)	12(100)	1156(97.3)
Three and more children	0(0)	15(1.1)	38(20.8)	174(16.4)	43(24.2)	210(22)	23(16.8)	135(13.9)	44(32.1)	269(29)	2(6.1)	49(4.7)	0(0)	32(2.7)
**Household characteristics**														
Type of toilet facility														

	Bangladesh		India		Pakistan		Kenya		Mali		Malawi		Tanzania	
	Prolonged diarrhoea (%) (n=76)	Acute Diarrhoea (%) (n=1355)	Prolonged diarrhoea (%) (n=183)	Acute Diarrhoea (%) (n=1063)	Prolonged diarrhoea (%) (n=178)	Acute Diarrhoea (%) (n=954)	Prolonged diarrhoea (%) (n=137)	Acute Diarrhoea (%) (n=973)	Prolonged diarrhoea (%) (n=137)	Acute Diarrhoea (%) (n=929)	Prolonged diarrhoea (%) (n=33)	Acute Diarrhoea (%) (n=1048)	Prolonged diarrhoea (%) (n=12)	Acute Diarrhoea (%) (n=1188)
Not improved	0(0)	7(0.5)	6(3.3)	19(1.8)	1(0.6)	7(0.7)	55(40.1)	343(35.3)	10(7.3)	75(8.1)	1(3)	8(0.8)	0(0)	5(0.4)
Improved	76(100)	1348(99.5)	177(96.7)	1044(98.2)	177(99.4)	947(99.3)	82(59.9)	630(64.7)	127(92.7)	854(91.9)	32(97)	1040(99.2)	12(100)	1183(99.6)
Water supply														
Not-piped	9(11.8)	133(9.8)	74(40.4)	429(40.4)	81(45.5)	405(42.5)	108(78.8)	794(81.6)	38(27.7)	249(26.8)	5(15.2)	252(24)	2(16.7)	75(6.3)
Piped	67(88.2)	1222(90.2)	109(59.6)	634(59.6)	97(54.5)	549(57.5)	29(21.2)	179(18.4)	99(72.3)	680(73.2)	28(84.8)	796(76)	10(83.3)	1113(93.7)
Type of floor														
Not-cemented	0(0)	64(4.7)	39(21.3)	220(20.7)	46(25.8)	179(18.8)	73(53.3)	439(45.1)	11(8)	34(3.7)	4(12.1)	235(22.4)	0(0)	29(2.4)
Cemented	76(100)	1291(95.3)	144(78.7)	841(79.3)	132(74.2)	775(81.2)	64(46.7)	534(54.9)	126(92)	895(96.3)	29(87.9)	813(77.6)	12(100)	1159(97.6)

Table S3. Anthropometric outcome and hospitalization of the study participants enrolled at seven sites during the study period by age stratification and intervention group

	Prolonged diarrhoea	Acute diarrhoea	*P*-value*	RR or RD (95% CI)	*P*-value
**Age 2-12m (N=4652)**	**(n=452)**	**(n=42000**			
Anthropometric outcome**					
90-d Δ LAZ, change, mean (SD)	-0.07±0.72	-0.22±0.66	<0.001	RD 0.07 (0.004– 0.13)	0.037
90-d Δ WAZ, change, mean (SD)	0.33±0.68	0.15±0.62	<0.001	RD 0.16 (0.10– 0.22)	<0.001
90-d Δ WHZ, change, mean (SD)	0.31±0.93	0.22±0.88	0.052	RD 0.16 (0.07– 0.24)	<0.001
Hospitalization***					
By day 90	30 (6.6)	211 (5.0)	0.141	RR 1.18 (0.81– 1.72)	0.383
**Age >12-24m (N=3614)**	**(n=304)**	**(n=3310)**			
Anthropometric outcome**					
90-d Δ LAZ, change, mean (SD)	-0.12±0.40	-0.13±0.50	0.674	RD -0.01 (-0.06– 0.05)	0.764
90-d Δ WAZ, change, mean (SD)	0.24±0.46	0.20±0.50	0.175	RD 0.05 (-0.004– 0.11)	0.069
90-d Δ WHZ, change, mean (SD)	0.42±0.71	0.37±0.72	0.299	RD 0.08 (-0.003– 0.16)	0.058
Hospitalization***					
By day 90	16 (5.3)	124 (3.8)	0.190	RR 1.30 (0.78– 2.17)	0.306
**Intervention or Azithromycin group (N=4131)**	**(n=377)**	**(n=3754)**			

Anthropometric outcome**					
90-d Δ LAZ, change, mean (SD)	-0.12±0.54	-0.16±0.60	0.233	RD -0.03 (-0.09–0.03)	0.332
90-d Δ WAZ, change, mean (SD)	0.25±0.59	0.19±0.58	0.057	RD 0.05 (-0.01– 0.11)	0.121
90-d Δ WHZ, change, mean (SD)	0.32±0.81	0.30±0.82	0.651	RD 0.07 (-0.01– 0.15)	0.101
Hospitalization					
By day 90	23 (6.1)	147 (3.9)	0.042	RR 1.31 (0.84– 2.04)	0.227

***** Used t-test for 90-d Δ LAZ, WAZ, WHZ and Chi-square for hospitalization

**Linear regression adjusted for age, maternal education, number of under 5 children in family, baseline z-score and sites

*** Log binomial regression adjusted for baseline z-score and sites

RD, risk difference; RR, relative risk; SD, standard deviation
